# A polygenic risk score modifies the cardiovascular risk associated with obstructive sleep apnea

**DOI:** 10.1093/sleepadvances/zpag037

**Published:** 2026-03-23

**Authors:** Christian W Thorball, Adrien Waeber, Geoffroy Solelhac, Flavia Hodel, Théo Imler, Grégory Heiniger, Roxane de La Harpe, Pedro Marques-Vidal, Peter Vollenweider, Jacques Fellay, Raphaël Heinzer

**Affiliations:** Precision Medicine Unit, Biomedical Data Science Center, Lausanne University Hospital (CHUV) and University of Lausanne, Lausanne, Switzerland; Center for Investigation and Research in Sleep (CIRS), Lausanne University Hospital (CHUV) and University of Lausanne, Lausanne, Switzerland; Center for Investigation and Research in Sleep (CIRS), Lausanne University Hospital (CHUV) and University of Lausanne, Lausanne, Switzerland; Precision Medicine Unit, Biomedical Data Science Center, Lausanne University Hospital (CHUV) and University of Lausanne, Lausanne, Switzerland; Center for Investigation and Research in Sleep (CIRS), Lausanne University Hospital (CHUV) and University of Lausanne, Lausanne, Switzerland; Center for Investigation and Research in Sleep (CIRS), Lausanne University Hospital (CHUV) and University of Lausanne, Lausanne, Switzerland; Department of Medicine, Internal Medicine, Lausanne University Hospital (CHUV) and University of Lausanne, Lausanne, Switzerland; Department of Medicine, Internal Medicine, Lausanne University Hospital (CHUV) and University of Lausanne, Lausanne, Switzerland; Department of Medicine, Internal Medicine, Lausanne University Hospital (CHUV) and University of Lausanne, Lausanne, Switzerland; Precision Medicine Unit, Biomedical Data Science Center, Lausanne University Hospital (CHUV) and University of Lausanne, Lausanne, Switzerland; School of Life Sciences, École Polytechnique Fédérale de Lausanne, Lausanne, Switzerland; Center for Investigation and Research in Sleep (CIRS), Lausanne University Hospital (CHUV) and University of Lausanne, Lausanne, Switzerland

**Keywords:** obstructive sleep apnea, polygenic risk score, cardiovascular risk, sleep disordered breathing, genetic, genome-wide association study

## Abstract

**Study Objectives:**

Obstructive sleep apnea (OSA) carries increased cardiovascular (CV) risk. However, this risk is not fully captured by the apnea–hypopnea index (AHI). We investigated whether a validated coronary artery disease polygenic risk score (CAD-PRS) refines CV risk assessment in OSA.

**Methods:**

We derived CAD-PRS using genome-wide genotyping data for 1379 participants of the CoLaus|HypnoLaus cohort who underwent polysomnography. Associations between OSA, CAD-PRS, clinical factors, and incident CV events were assessed using multivariable Cox proportional hazards models. Risk stratification improvement was assessed with reclassification analyses compared to clinical risk scores (SCORE2/SCORE2-OP).

**Results:**

During 7.2 years of median follow-up, 100 participants experienced CV events. A significant interaction between OSA and CAD-PRS was observed (p=.013). The effect of OSA on CV risk differed across PRS categories. In the intermediate genetic-risk group (CAD-PRS quintiles 2–4), OSA patients (AHI ≥15/h) had a markedly higher CV risk compared to non-OSA (HR[95% CI]: 2.68[1.54–4.66]), whereas OSA did not significantly increase CV risk in either the low or high PRS strata. The complete model with OSA, CAD-PRS and their interaction allowed a significant reclassification (Net Reclassification Index 0.171, p=.014) compared to SCORE2/SCORE2-OP and 52% of individuals at intermediate risk were reclassified as low or high CV risk.

**Conclusions:**

In this population-based cohort, a CAD-PRS was associated with CV risk stratification in individuals with OSA. The impact of OSA on CV risk was greatest in individuals with intermediate genetic risk. Adding CAD-PRS and OSA to SCORE2 was associated with improved model performance and reclassification, supporting more precise CV risk assessment in OSA.

## Graphical Abstract

**Graphical Abstract:**
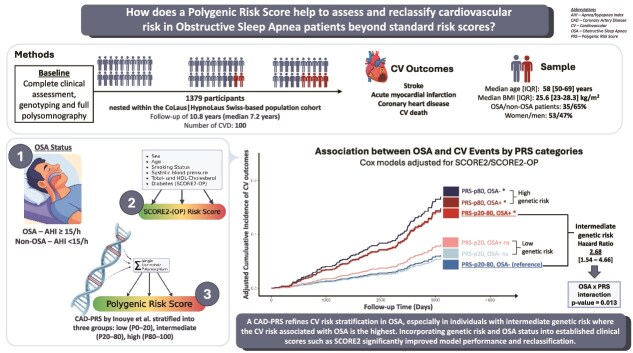

Statement of Significance Obstructive sleep apnea is common, however the apnea–hypopnea index alone does not adequately capture associated cardiovascular risk. In this study, we evaluated whether a polygenic risk score for coronary artery disease can refine cardiovascular risk assessment in people with sleep apnea. Both sleep apnea and genetic risk were independently associated with incident cardiovascular events beyond standard clinical risk scores. Importantly, we found a significant interaction: excess cardiovascular risk related to moderate-to-severe sleep apnea was mainly confined to individuals with an intermediate genetic risk, while those at low or very high genetic risk were less affected. These findings support the potential value of incorporating genetic information to enable more nuanced and personalized cardiovascular risk assessment in sleep apnea.

## Introduction

Obstructive sleep apnea (OSA) is characterized by repetitive episodes of partial or complete airway obstruction during sleep and affects up to 49% of men and 23% of women over 40 years old [1]. OSA has been associated with various adverse health outcomes, including daytime sleepiness, increased risk of hypertension, stroke, heart failure, diabetes and metabolic syndrome [2–5]. Gold standard sleep recordings allow to accurately measure each of these respiratory events. Traditionally, OSA severity is defined according to the apnea-hypopnea index (AHI) by counting the number of respiratory events per hour of sleep [6]. Standard and rather arbitrary thresholds are used as an indication for treatment. However, randomized controlled trials suggest that the AHI may not be specific enough to single out patients with OSA at risk for developing apnea-associated cardiovascular diseases (CVD) and who would benefit from treatment [7–9]. This raises the need for more specific markers for cardiovascular (CV) risk stratification in OSA [10].

In the last decade, several studies have tried to target patients at risk of apnea-associated CVD using specific polysomnographic biomarkers [11] such as hypoxic burden [12], respiratory events lengths [13], sleep apnea-related pulse rate response [14, 15], pulse wave amplitude drop index [16] or subjective complaints such as excessive daytime sleepiness [17] or insomnia complaints [18, 19]. However, even if sleep apnea is an independent cardiovascular risk factor, it remains essential to consider the patient’s overall cardiovascular risk profile, using validated global CV risk assessment tools, such as SCORE2/SCORE2-OP [20, 21] which have been developed to predict 10 years risk of first-onset CVD in European populations. In addition, integrating polygenic risk scores (PRS) and other genetic information holds promise for further enhancing cardiovascular risk stratification among patients with OSA, by capturing genetic susceptibility beyond traditional clinical factors [22].

In that view, genome-wide association studies have enabled the development of PRS, which combine the effects of many genetic variants throughout the genome to effectively quantify the individual genetic predisposition to CVD [23]. Applying PRS to high-risk groups such as OSA patients is still debated. For example, in the UK Biobank, the effect of OSA on CAD risk did not change depending on a person’s CAD-PRS, but significant interactions emerged when using scores focused on specific pathways of intermittent hypoxemia [24]. This suggests that the interaction between genetic susceptibility and OSA is complex and likely non-linear, potentially affecting specific biological pathways rather than acting uniformly across all risk levels. Instead, it may involve specific biological pathways, meaning that a true interaction could be obscured when using broad, undifferentiated PRS models.

Therefore, the primary aim of the present study was to assess whether a genome-wide CAD-PRS enhances cardiovascular risk prediction in OSA and to specifically investigate the potential for a non-linear interaction between genetic predisposition and OSA severity. The secondary aim was to investigate whether integrating PRS within a validated clinical CV risk score (SCORE2/SCORE2-OP) improves risk reclassification in OSA patients. Identifying high-risk OSA patients using genetic testing may enable more personalized management and intensifying control of other cardiovascular risk factors.

## Materials and Methods

### Ethics statement

The institutional Ethics Committee of the University of Lausanne, which afterwards became the Ethics Commission of Canton Vaud (www.cer-vd.ch) approved the CoLaus|PsyCoLaus study (project number PB_2018-00038, reference 239/09). All participants gave their signed informed consent before entering the study.

### Participants

CoLaus**|**PsyCoLaus is a prospective cohort study aiming to evaluate the prevalence and associations of mental disorders and cardiovascular risk factors in the community of Lausanne, Switzerland and to identify genetic determinants and mechanisms involved in their association. The sample, composed of 6733 people, was randomly selected from the 35 to 75-year-old residents of the city of Lausanne, Switzerland, from 2003 to 2006 according to the civil register and 4791 subjects participated in the genetic analysis [25].

All participants underwent thorough physical and psychiatric evaluations at baseline and at three follow-up visits (every 5 years approximately). During first follow-up, 2162 participants (aged 45–80 years) took part in the sleep sub-cohort HypnoLaus, and underwent an unattended overnight full polysomnography (PSG) at home between 2009 and 2013 [1]. Prospective data on cardiovascular events were obtained from the third follow-up of the CoLaus|PsyCoLaus study (between 2018 and 2021).

**Figure 1:**
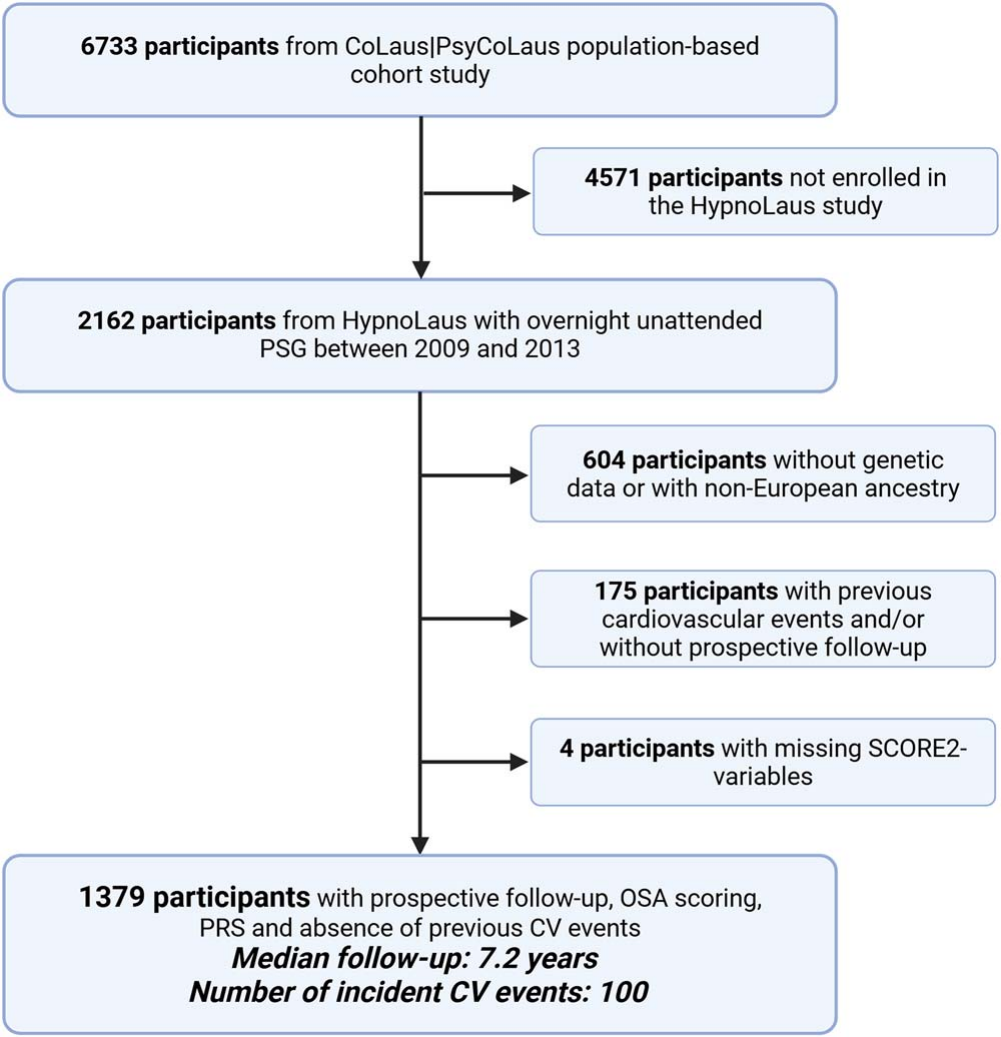
Study flow chart. Flow chart showing selection of participants from the CoLaus|PsyCoLaus cohort and the HypnoLaus sleep sub-study to the final analytic sample with polysomnography, CAD-PRS and follow-up. Participants with missing PSG or genetic data, prior cardiovascular events, non-European ancestry or no follow-up were excluded. The median follow-up was 7.2 years, during which 100 incident CV events occurred. Abbreviations: PSG, polysomnography; OSA, obstructive sleep apnea; CAD-PRS, coronary artery disease polygenic risk score; CV, cardiovascular.

Inclusion criteria were: participation in the HypnoLaus study with valid PSG data, availability of genotyping data, no history of CVD at the time of PSG, availability of follow-up data, and European ancestry. As illustrated in Figure 1, a total of 1379 individuals met these criteria and were included in the final analysis. Participants of non-European ancestry were excluded to ensure the validity of the CAD-PRS, which was developed and validated in populations of European descent.

### Cardiovascular outcomes

The **primary outcome** was incident cardiovascular (CV) events, defined as a composite of cardiovascular death, stroke (definite or probable), acute myocardial infarction (AMI, definite or probable), and coronary heart disease (CHD, definite or probable).

For sensitivity analyses, we considered three alternative definitions of cardiovascular outcome:

(1) **Hard CV outcomes (including transient ischemic attack [TIA]):** cardiovascular death, stroke (definite or probable) and TIA (excluding amaurosis fugax and transient global amnesia), AMI (definite or probable), and CHD (definite or probable).
(2) **Hard CV outcomes (excluding TIA):** cardiovascular death, stroke (definite or probable; excluding TIA, amaurosis fugax and transient global amnesia), AMI (definite or probable), and CHD (definite or probable).
(3) **Pure hard CV outcomes:** cardiovascular death, definite stroke, definite AMI, and definite CHD.

Events were identified through medical records, hospital databases, death registries, and follow-up interviews, and were independently adjudicated by at least two specialists (cardiologists, neurologists, or internists) using predefined international criteria, as described by Beuret et al. [26]

Time-to-event was calculated from the date of PSG to the first incident CV event or the last follow-up, whichever occurred first.

### Clinical assessment

Blood samples were collected following overnight fasting for biochemical assays, including glucose, insulin and lipid profiles as previously described [25]. Smoking status, alcohol consumption, and use of medications were reported, and sleep questionnaires were collected (Epworth Sleepiness Scale [ESS], Pittsburgh Sleep Quality Index [PSQI]) [27, 28].

Anthropometric measurements were performed by trained observers with standard techniques. Body weight, height, and waist circumference were measured with participants standing without shoes in light clothes. Body mass index (BMI) was calculated as body mass in kg divided by the square of the patient’s height in meters.

BP was measured in triplicate on the left arm and values averaged between the last two readings. Arterial hypertension was defined as a systolic BP (SBP) ≥ 140 mmHg and/or a diastolic BP (DBP) ≥ 90 mmHg or, current use of antihypertensive medication.

### Polysomnography

The participants performed a full night PSG at home (Titanium, Embla® Flaga, Reykjavik, Iceland). PSG were performed according to the American Academy of Sleep Medicine (AASM) 2007 recommendations and included: EEG leads (F3, F4, C1, C2, O1, and O2, 256 Hz sampling rate), electrooculography (EOG, left and right), electromyography (EMG, chin and anterior tibialis muscle), electrocardiography (ECG, one lead), oxygen saturation (SpO_2_), airflow (nasal cannula), abdominal and thoracic respiratory efforts, snoring, and body position. PSG data were visually scored according to the AASM 2007 guidelines [29].

Two trained sleep technicians scored polysomnographic recordings using Somnologica software (version 5.1.1, Embla Flaga, Reykjavik, Iceland). An expert sleep clinician reviewed every recording, and a second sleep expert did random quality checks. We defined apnea as a drop of at least 90% of airflow from baseline lasting 10 s or longer. We scored hypopnea events with 2012 AASM criteria [30] (≥30% drop of airflow lasting at least 10 s with either an arousal or ≥ 3% oxygen saturation drop). We reported the average number of apnea and hypopnea events per hour of sleep (AHI). OSA status was defined as a binary variable (non-moderate/severe: AHI <15 events/h vs. moderate/severe: AHI ≥15 events/h).

### Genotyping and imputation

DNA was extracted from whole blood and samples were genotyped using the BB2 GSK-customized Affymetrix Axiom Biobank array as described earlier [25, 31]. Variants and samples with excessive missingness above 5% or variants deviating from Hardy–Weinberg equilibrium (HWE, p<10e-7) were excluded prior to imputation.

Imputation was performed in two separate runs with the Sanger Imputation Service [32], using Positional Burrows Wheeler Transform [33] with variants first phased using EAGLE2 (v2.0.5) [34] and then imputed using the merged 1000 Genomes Project phase 3 plus UK10K reference panel and subsequently the Haplotype Reference Consortium r1.1 reference panel. The two imputed datasets were then merged, retaining only high-quality single nucleotide polymorphisms with an imputation information score (INFO) > 0.8. In cases where variants in both datasets had an INFO score above this threshold, the variant from the dataset with the highest INFO score was retained. For the final dataset, rare variants (minor allele frequency below 1%), variants with high missingness (above 10%) or with a deviation from HWE (p<10e-7) were removed prior to calculating the PRS. Assessment of the presence of potential cryptic related or duplicate samples was performed using KING (v2.2.6) [35]. Ancestry was determined using principal component analysis with PLINK (v2.00a3LM) [36] together with the 1000 Genomes Project reference panel.

### Polygenic risk score

The CAD-PRS was derived using PRSice (v2.3.5) [37] based on the previously validated CAD-PRS by Inouye et al. [23], with information on the included variants and their respective effect sizes obtained from the Polygenic Score Catalog [38] (PGS000018). This PRS was developed using data from the UK Biobank, with CAD defined as diagnosis of fatal or non-fatal myocardial infarction (MI), or history of percutaneous transluminal coronary angioplasty, or of coronary artery bypass grafting. In total, 1 357 679 of 1 745 179 variants from the original CAD-PRS were successfully matched and included in this study. The resulting raw PRS scores were standardized (i.e. scaled to a mean of 0 and a standard deviation of 1) prior to analysis.

### SCORE2/SCORE2-op

SCORE2/SCORE2-OP risk scores were calculated at time of PSG to assess 10-year cardiovascular risk using the low-risk model as applicable to Switzerland using the RiskScorecvd (v0.3.1) R package [39]. Categories were defined by the published SCORE2/SCORE2-OP age-specific 10-year risk thresholds for fatal and non-fatal CVD (low, intermediate, high risk: <2.5%, 2.5–7.5%, ≥7.5% for individuals <50 years; <5%, 5–10%, ≥10% for 50–69 years; and < 7.5%, 7.5–15%, ≥15% for ≥70 years) [20, 21].

### Statistical analyses

Baseline characteristics of the study population were summarized using medians and interquartile ranges (IQR) for continuous variables and frequencies with percentages for categorical variables. Differences in baseline characteristics between participants with and without incident CV events were assessed using the Wilcoxon rank-sum test for continuous variables and Pearson’s Chi-squared test for categorical variables.

The primary outcome was first incident CV event. The association between risk factors and incident CV events was evaluated using Cox proportional hazards models. The proportionality of hazards assumption was assessed graphically using log–log survival plots and formally tested using Schoenfeld residuals.

To evaluate the independent and combined predictive value of clinical risk, genetic risk, and OSA, a series of nested Cox models was constructed. The base model included the SCORE2/SCORE2-OP risk score as a continuous variable. The CAD-PRS was included as a categorical variable. We categorized CAD-PRS into three strata: Low (bottom quintile, <20%), Intermediate (quintiles 2–4, 20%–80%), and High (top quintile, >80%). The Intermediate group served as the reference category. This stratification was chosen to maximize statistical power by establishing a large, stable reference group, and to specifically capture non-linear risk trajectories where clinical utility is concentrated in the tails of the genetic distribution, as previously described by Mega et al. [40] and Marston et al. [41] All models were adjusted for population stratification by including the top five principal components of the genotyping matrix as covariates.

To test for a statistical interaction between genetic risk and OSA, an interaction term (PRS group × OSA status) was added to the multivariable Cox model containing SCORE2, PRS, and OSA main effects. The significance of the interaction was determined using the likelihood ratio test (LRT).

Model performance was assessed using several metrics. The improvement in model fit for nested models was quantified using the LRT p-value, Akaike Information Criterion (AIC), and Bayesian Information Criterion (BIC). Discriminatory ability was evaluated using Harrell’s C-index. The proportion of variance explained was estimated using Nagelkerke’s pseudo-R2. Calibration was assessed numerically using the optimism-corrected calibration slope (200 bootstrap resamples) via the rms (v8.1-0) R package [42]. Visual calibration for the 10-year prediction horizon was evaluated using the riskRegression (v2025.09.17) R package [43]. Smoothed calibration curves were generated using nearest neighbor estimation with jackknife pseudo-values to account for censoring, superimposed with a density plot to illustrate the distribution of predicted risks.

To evaluate clinical utility and risk stratification improvement, reclassification analyses were performed. We calculated the Integrated Discrimination Improvement (IDI) and the continuous Net Reclassification Improvement (NRI) for models with the addition of PRS, OSA, and their interaction plus the five first principal components, compared to the base model using the survIDINRI R package [44]. The categorical NRI was determined using established age-dependent risk strata for SCORE2/SCORE-OP, and a bootstrap procedure with 1000 replicates was employed to derive its 95% confidence interval and p-value.

Adjusted cumulative incidence curves were generated from the final Cox model to visualize the probability of incident CV events over time across different strata of PRS and OSA status.

All statistical analyses were conducted using R (v4.4.2).

## Results

After excluding 783 HypnoLaus participants due to missing genotyping data, non-European ancestry, prevalent CVD at baseline, or other missing data points, 1379 individuals were included in the analysis, of which 100 individuals experienced at least one adjudicated definite or probable CV event during follow-up (Figure 1). Baseline characteristics at the time of PSG are shown in Table 1.

**Table 1:** Patient Characteristics according to incident cardiovascular events. Baseline characteristics of the 1379 participants with polysomnography, genetic data and follow-up, overall and according to the occurrence of incident cardiovascular events during follow-up

			Cardiovascular event		
Variable	N	**Overall** ***n* = 1379** ^*^	**No** ***n* = 1279** ^*^	**Yes** ***n* = 100** ^*^	**p-value** ^**^
OSA	1379				<0.001
No		891 (65%)	846 (66%)	45 (45%)	
Yes		488 (35%)	433 (34%)	55 (55%)	
SCORE2 category	1379				<0.001
Low risk		768 (56%)	738 (58%)	30 (30%)	
Moderate risk		472 (34%)	430 (34%)	42 (42%)	
High risk		139 (10%)	111 (8.7%)	28 (28%)	
Sex	1379				0.1
Female		731 (53%)	686 (54%)	45 (45%)	
Male		648 (47%)	593 (46%)	55 (55%)	
Age (y)	1379	58 (50, 69)	58 (49, 68)	68 (57, 74)	<0.001
BMI (kg/m^2^)	1373	25.6 (23.0, 28.3)	25.5 (23.0, 28.3)	26.3 (24.0, 28.8)	0.031
Unknown		6	4	2	
Current smoker	1379				0.26
No		1133 (82%)	1055 (82%)	78 (78%)	
Yes		246 (18%)	224 (18%)	22 (22%)	
Diabetes	1379				<0.001
No		1261 (91%)	1182 (92%)	79 (79%)	
Yes		118 (8.6%)	97 (7.6%)	21 (21%)	
Systolic BP (mmHg)	1379	125 (114, 138)	125 (114, 137)	135 (122, 147)	<0.001
Diastolic BP (mmHg)	1379	78 (71, 85)	78 (71, 85)	78 (72, 85)	0.64
Hypertension	1379				<0.001
No		823 (60%)	786 (61%)	37 (37%)	
Yes		556 (40%)	493 (39%)	63 (63%)	
Dyslipidemia	1379				0.038
No		1030 (75%)	964 (75%)	66 (66%)	
Yes		349 (25%)	315 (25%)	34 (34%)	
Statins	1379				0.003
No		1175 (85%)	1100 (86%)	75 (75%)	
Yes		204 (15%)	179 (14%)	25 (25%)	
HDL Cholesterol (mmol/L)	1379	1.60 (1.30, 1.90)	1.60 (1.30, 1.90)	1.50 (1.25, 1.80)	0.037
Total Cholesterol (mmol/L)	1379	5.70 (5.00, 6.40)	5.70 (5.00, 6.40)	5.75 (5.05, 6.45)	0.89
Weekly alcohol consumption (units)	1379	4 (1, 10)	4 (2, 10)	4 (1, 10)	0.46
AHI (events/h)	1379	10 (4, 21)	9 (4, 20)	17 (8, 30)	<0.001
ODI-3%	1379	10 (4, 19)	9 (4, 18)	17 (9, 28)	<0.001
Total sleep time (min)	1379	405 (359, 449)	405 (359, 448)	402 (353, 449)	0.78
Sleep efficiency (%)	1379	88 (80, 93)	88 (80, 93)	82 (75, 90)	<0.001
Mean SpO_2_	1379	94.20 (93.10, 95.30)	94.30 (93.20, 95.40)	93.60 (92.05, 94.40)	<0.001
PWADi	1369	52 (38, 66)	53 (39, 66)	45 (29, 60)	0.001
Unknown		10	10	0	

Abbreviations: OSA: obstructive sleep apnea; SCORE2: Systematic COronary Risk Evaluation 2; BMI: body mass index; BP: blood pressure; HDL: high-density lipoprotein; AHI: apnea–hypopnea index; ODI-3%: oxygen desaturation index (3% desaturation threshold); SpO₂: peripheral oxygen saturation by pulse oximetry; PWADi: pulse wave amplitude drops index.

^*^Median (IQR) or frequency (%).

^**^p-values derived from Pearson’s chi-squared test for categorical variables or Wilcoxon rank-sum test for continuous variables.

Participants who suffered a CV event were more likely to have OSA, were older and more frequently diabetic. They also had higher BMI, higher systolic blood pressure and higher HDL cholesterol levels. They also exhibited more severe sleep-disordered breathing, characterized by higher AHI, higher oxygen desaturation index at 3% (ODI-3%), lower mean oxygen saturation, poorer sleep efficiency, and a lower pulse wave amplitude drop index (PWADi). Baseline characteristics of participants according to OSA status are shown in Table S1 and by genetic risk in Table S2.

### CAD PRS is an independent risk factor for CVD among sleep apnea patients

We first evaluated whether the addition of the CAD-PRS to the validated SCORE2/SCORE2-OP algorithm improved the prediction of incident CV events among participants with and without moderate-to-severe OSA.

**Figure 2:**
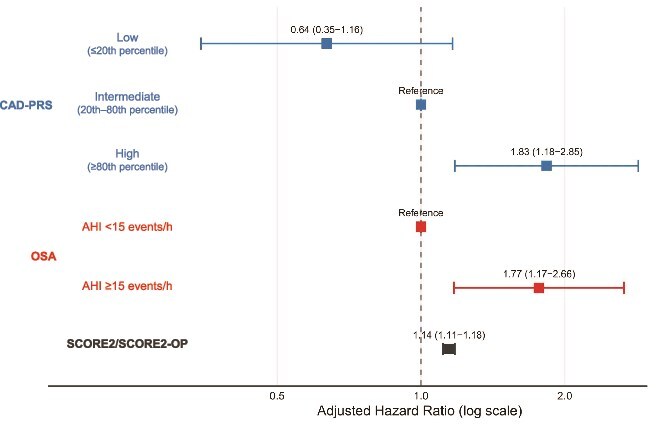
Forest plot of CAD-PRS, OSA and SCORE2 in relation to incident CV events. Forest plot showing adjusted hazard ratios and 95% confidence intervals for incident CV events from a cox model including SCORE2/SCORE2-OP, CAD-PRS, and OSA. CAD-PRS is modeled in three categories: Low (≤20th percentile), intermediate (20th–80th percentile, reference) and high (≥80th percentile). OSA is modeled as AHI ≥15 versus <15 events/h (reference). SCORE2/SCORE2-OP is entered as a continuous variable.

In Cox models adjusted for SCORE2/SCORE2-OP, both CAD-PRS and moderate-to-severe OSA were independently associated with CV risk (Figure 2). Using the lowest 20% of the CAD-PRS distribution as the reference, participants with PRS between the 20th and 80th percentile had a 1.57-fold higher risk of CV events (HR 1.57, 95% CI 0.86–2.89, p-value = 0.14), and those in the top 20% had an almost threefold higher risk (HR 2.88, 95% CI 1.50–5.53, p-value = 0.001). Similarly, moderate-to-severe OSA was associated with a 1.77-fold increased risk of CV events (HR 1.77, 95% CI 1.17–2.66, p-value = 0.007) compared with participants without OSA, independently of SCORE2. Each one-unit increase in SCORE2 was associated with a 14% higher risk of CV events (HR 1.14, 95% CI 1.11–1.18, p-value <0.001).

### Interaction between OSA and CAD-PRS

**Figure 3:**
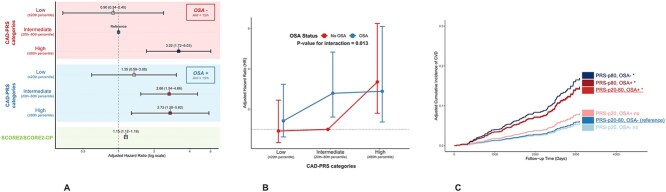
Combined effect of CAD-PRS and OSA on CV risk. (A) Forest plot showing adjusted hazard ratios (HRs) and 95% confidence intervals (CIs) for incident cardiovascular (CV) events according to CAD-PRS categories, stratified by OSA status. CAD-PRS is grouped as low (≤20th percentile), intermediate (20th–80th percentile) and high (≥80th percentile), separately for participants without OSA (AHI <15 events/h) and with moderate-to-severe OSA (AHI ≥15 events/h). (B) Interaction plot illustrating the adjusted HRs (with 95% CIs) for CV events across CAD-PRS categories in participants with and without OSA, with the corresponding p-value for the OSA × CAD-PRS interaction. (C) Adjusted cumulative incidence curves for CV events according to combined CAD-PRS/OSA groups, using the intermediate-risk, OSA-negative group as the reference. All HRs are derived from cox model adjusted for SCORE2/SCORE2-OP.

A significant non-linear interaction was observed between OSA severity and CAD-PRS (p-interaction = 0.013). When stratified by PRS categories, only individuals with intermediate genetic risk (PRS 20th–80th percentile) had a significantly higher CV risk associated with OSA (AHI ≥15 events/h) with a HR of 2.68 (95% CI 1.54–4.66, p-value <0.001). In contrast, for participants at the extremes of the PRS distribution, the presence of OSA did not significantly modify CV risk. In the lowest PRS category, HR for OSA was 1.37 (95% CI 0.43-4.36, p-value = 0.59). In the highest PRS category, HR for OSA was 0.99 (95% CI 0.44-2.22, p-value = 0.98). Full model, non-linear interaction and adjusted cumulative incidence curve are shown in Figures 3A–C.

### Sensitivity analysis

Because the definition of CV outcomes may influence the observed associations, we conducted sensitivity analyses using progressively stricter outcome definitions and further adjusted the models for medication use (antidiabetic, antihypertensive, and lipid-lowering therapies). Across all outcome definitions (hard CV outcomes including TIA, hard CV outcomes excluding TIA, and pure hard CV outcomes, as described in methods) participants with moderate-to-severe OSA in the mid-range PRS category (PRS quintiles 2–4) consistently exhibited a significantly higher risk of incident CVD compared with those without OSA, consistent with the main analysis (Figures S1, S2, and S3).

The interaction between OSA and PRS remained significant for hard CV outcomes including TIA (p-value = 0.021) and excluding TIA (p-value = 0.045) and showed a similar trend for pure hard CV outcomes (p-value = 0.108) (Figures S1, S2, and S3). Further adjustment for medication use yielded essentially unchanged results, with significant interactions for any CVD (p-value = 0.016) and hard CVD (p-value = 0.048) (Figures S4 and S5).

In a sensitivity analysis excluding participants treated with continuous positive airway pressure (CPAP) (*n* = 114), the interaction signal was strengthened. The linear interaction between continuous PRS and continuous AHI became statistically significant (p-value = 0.048), with a negative interaction coefficient (-0.012) indicating a “ceiling” or saturation effect where the relative impact of OSA diminishes as genetic risk becomes the dominant driver. This non-linear relationship was further confirmed by restricted cubic spline modeling (Figure S6), showing distinct risk divergence in the intermediate genetic risk range. These findings highlight the robustness of the observed interaction between OSA status and genetic risk, particularly among individuals in the intermediate PRS category.

### Model performances and improvement in risk classification

Global model performance metrics are summarized in Table 2. The base model achieved a C-index of 0.768 and a Nagelkerke’s R^2^ of 0.088. Adding the PRS improved discrimination (C-index 0.781, R^2^ = 0.104; LRT p-value = 0.035), while the inclusion of OSA also significantly enhanced model fit (C-index 0.78, R^2^ = 0.112; LRT p-value = 0.004). The combined model incorporating PRS, OSA and their interaction term achieved the highest overall explanatory power (C-index 0.782, R^2^ = 0.118; LRT p-value = 0.001), although incremental gains in discrimination were limited. All models showed good calibration (Figure S7).

**Table 2:** Comparison of Cox models for prediction of cardiovascular events. Comparison of Cox proportional hazards models for prediction of incident cardiovascular events. The SCORE2 model includes SCORE2/SCORE2-OP only; SCORE2 + PRS additionally includes the CAD-PRS; SCORE2 + OSA additionally includes moderate-to-severe OSA; SCORE2 + PRS + OSA includes both CAD-PRS and OSA; and SCORE2 + OSA + PRS + PRS * OSA further includes the interaction term between CAD-PRS and OSA. Lower AIC/BIC and higher Nagelkerke’s R^2^ and C-index indicate better model performance. LRT p-values compare each extended model with the SCORE2 model

Cox Model	AIC	BIC	Nagelkerke’s R2	RMSE	C-index	LRT p-value
**SCORE2**	1324.6	1329.8	0.088	0.273	0.768	-
**SCORE2 + PRS**	1323.5	1365.4	0.104	0.276	0.781	0.035
**SCORE2 + OSA**	1319.3	1329.8	0.096	0.272	0.756	0.007
**SCORE2 + PRS + OSA**	1318.1	1365.1	0.112	0.276	0.780	0.004
SCORE2 + OSA + PRS + PRS * OSA	1316.0	1373.5	0.118	0.273	0.782	0.001

Abbreviations: AIC: Akaike Information Criterion; BIC: Bayesian Information Criterion; RMSE: root-mean-square error; C-index: Harrell’s concordance index; LRT: likelihood ratio test.

To further assess the clinical relevance of these improvements, we examined reclassification metrics when compared to SCORE2 as a continuous variable (Table 3). The PRS yielded only modest gains in discrimination (IDI 0.020, p-value = 0.016) without significant improvement in net reclassification. In contrast, OSA provided substantial incremental value (IDI 0.027, p-value <0.001; continuous NRI 0.450, p-value = 0.068). The combined model including both PRS and OSA, and particularly the model with their interaction term, achieved the strongest reclassification improvements (IDI 0.038 p-value = 0.004 and 0.057 p-value ≤0.001, respectively), with significant increases in continuous NRI and median risk score improvement.

**Table 3:** Improvements in discrimination and reclassification after adding CAD-PRS and OSA. IDI and continuous NRI for models adding CAD-PRS and OSA to SCORE2/SCORE2-OP for 10-year cardiovascular risk prediction (1000 bootstrap resamplings). Results are shown for models including SCORE2 plus PRS, SCORE2 plus OSA, SCORE2 plus PRS and OSA, and SCORE2 plus PRS × OSA interaction. A positive continuous NRI indicates that the new model more often reclassifies risk in the correct direction (higher for cases, lower for controls). Positive categorial NRI indicates the model correctly moves cases to higher risk categories. A positive IDI indicates improved separation between cases and controls by the new model

Cox Model	Est.	Lower	Upper	p-value
**SCORE2 + PRS**				
IDI	0.020	0.006	0.060	0.016
Continuous NRI	0.246	-0.152	0.564	0.194
Median improvement in risk score	0.001	-0.005	0.030	0.276
Categorical NRI	0.029	-0.094	0.153	0.646
**SCORE2 + OSA**				
IDI	0.027	0.009	0.070	0.000
Continuous NRI	0.450	-0.061	0.638	0.068
Median improvement in risk score	0.023	-0.001	0.055	0.054
Categorical NRI	-0.032	-0.139	0.068	0.504
**SCORE2 + PRS + OSA**				
IDI	0.038	0.020	0.084	0.004
Continuous NRI	0.662	0.191	0.740	0.010
Median improvement in risk score	0.029	0.006	0.064	0.012
Categorical NRI	0.071	-0.042	0.197	0.240
SCORE2 + OSA + PRS + PRS * OSA				
IDI	0.057	0.035	0.116	0.000
Continuous NRI	0.653	0.328	0.774	0.004
Median improvement in risk score	0.058	0.022	0.099	0.000
Categorical NRI	0.171	0.042	0.305	0.014

Abbreviations: IDI: integrated discrimination improvement; Cont. NRI: continuous net reclassification improvement; Cat. NRI: categorical net reclassification improvement.

When using SCORE2/SCORE2-OP categories, the extended model significantly improved patient reclassification across all age groups with a total categorical NRI: 0.1707 (95% CI: 0.0420 to 0.3047, p-value = 0.014) and a NRI for Events: 0.0300 (95% CI: -0.0893 to 0.1604), NRI for Non-Events: 0.1407 (95% CI: 0.1015 to 0.1796). With SCORE2/SCORE2-OP, 915 of the 1379 participants were classified as moderate risk, where the full model reclassified 476 of these 915 (which represents 52.02%) in the low and high-risk group (*n* = 326 and 150, respectively).

## Discussion

In this large, prospective, population-based cohort, we observed three major findings. First, moderate-to-severe OSA (AHI ≥ 15 events/h) was linked to a higher incidence of adjudicated CVD events, especially in individuals with intermediate genetic susceptibility (PRS quintiles 2–4) highlighting a clinically relevant interaction between OSA and genetic predisposition. Second, integrating OSA, CAD-PRS and their interaction into the SCORE2/SCORE2-OP algorithms was associated with significantly improved cardiovascular risk discrimination and reclassification beyond clinical risk factors alone. Finally, the CAD-PRS was independently associated with the occurrence of adjudicated CV events over a 10-year follow-up among participants with and without OSA, supporting the validity of this PRS in our study population.

### Interaction between OSA and genetic risk

In this sample, the excess cardiovascular risk associated with moderate-to-severe OSA was most evident in participants at intermediate genetic risk, who represented 60% of the cohort. At the extremes of genetic risk, OSA severity had little incremental value: participants with low PRS remained at low risk regardless of OSA, while those with high PRS were already at very high risk. These findings suggest that OSA severity has additional discriminatory value particularly among individuals with intermediate genetic risk, and may indicate a potential ceiling effect in risk accumulation, where the distinct contribution of OSA becomes less discernible in the presence of an extreme polygenic risk profile. The model performance and improvement in risk classification indicate that while the PRS contributes modestly on its own, OSA and its interaction with genetic risk seems to provide the greatest incremental predictive value.

### Clinical implications

Our findings have direct clinical relevance. Incorporating CAD-PRS into existing clinical risk scores could help clinicians identify OSA patients at highest cardiovascular risk. If externally validated, these findings could support more aggressive prevention strategies, such as stricter control of modifiable risk factors, prioritization for follow-up and earlier initiation of CPAP therapy.

There is also evidence that knowledge of genetic risk can motivate positive health behaviors and treatment adherence [45, 46]. Thus, CAD-PRS may not only refine risk prediction but also enhance patient engagement, which is crucial in OSA management where CPAP adherence remains a major challenge. However, future studies should investigate whether CAD-PRS can predict the cardiovascular benefit of CPAP therapy, as previously shown for statins and other preventive interventions in high genetic risk patients [47].

Importantly, patients with moderate OSA (AHI 15–30 events/h) represent a particularly challenging group for treatment decisions, especially when they are minimally symptomatic or asymptomatic. In this population, the added value of CAD-PRS in identifying individuals at higher CV risk is encouraging, as it may help refine therapeutic decisions and supports further studies evaluating whether CPAP effectively reduces cardiovascular events in genetically high-risk moderate OSA patients.

Beyond our study, the clinical feasibility of integrating genetic analysis into routine care is improving rapidly. Although cost-effectiveness studies specifically tailored to OSA populations are currently lacking, evidence from the broader cardiovascular field is encouraging [48]. A recent American Heart Association scientific statement highlights the utility of PRS in enhancing risk prediction and facilitating shared decision-making, particularly for individuals in intermediate clinical risk categories where management decisions are often challenging [49]. Furthermore, the economic viability of genetic assessment should be viewed through a cumulative lens. Because a single genotyping assay or whole genome sequencing run yields raw data applicable to multiple disease traits and pharmacogenetics throughout a patient’s life, the long-term utility likely outweighs the initial costs, which continue to decline. Recent primary care trials have also demonstrated that incorporating PRS into cardiovascular risk checks is feasible and widely accepted by both practitioners and patients, supporting the potential for similar implementation in sleep medicine [50].

### Strengths and limitations

The strengths of our study include its prospective design, long follow-up, rigorous adjudication of CV events, comprehensive phenotyping with home-based PSG, and use of a validated CAD-PRS.

However, several limitations merit consideration. The number of cardiovascular events was limited, which may have reduced power for subgroup and interaction analyses. The study population consisted exclusively of individuals of European ancestry, limiting generalizability to other ethnicities. CPAP treatment and other OSA interventions during follow-up were not systematically recorded and could have influenced the observed associations. Finally, this study lacks external validation for further generalization.

### Future directions

Our findings pave the way for further research integrating genetic information into the management of OSA. Larger, multi-ethnic cohorts are needed to validate these observations, and randomized studies should test whether CAD-PRS-guided strategies improve cardiovascular outcomes among OSA patients. In addition, evaluating whether CAD-PRS modifies the benefit of CPAP therapy or other interventions could inform more personalized treatment approaches.

## Conclusion

In this prospective cohort, a CAD-PRS was independently associated with incident CV events and interacted with OSA status to refine cardiovascular risk stratification. The excess risk conferred by moderate-to-severe OSA was particularly evident among individuals with intermediate genetic risk, who represent the majority of the population. Incorporating genetic risk and OSA status into established clinical scores such as SCORE2 was associated with significant improvement of model performance and reclassification, highlighting the potential of CAD-PRS to guide cardiovascular risk assessment in OSA patients.

## Supplementary Material

zpag037_Supplemental_Files

## Data Availability

The data of CoLaus|PsyCoLaus used in this study cannot be fully shared as they contain potentially sensitive personal information on participants. According to the Ethics Committee, sharing these data would be a violation of the Swiss legislation concerning privacy protection. However, coded individual-level data that do not allow researchers to identify participants are available upon request to researchers who meet the criteria for data sharing (CHUV, Lausanne, Switzerland). Any researcher affiliated with a public or private research institution who complies with the CoLaus|PsyCoLaus standards can submit a research application to research.colaus@chuv.ch or research.psycolaus@chuv.ch. Detailed instructions for gaining access to the CoLaus|PsyCoLaus data used in this study are available at www.colaus-psycolaus.ch/professionals/how-to-collaborate/.
