# Supplementary material for: A polygenic risk score modifies the cardiovascular risk associated with obstructive sleep apnea
Source: Sleep Adv. 2026 Mar 23;7(2):zpag037. doi: 10.1093/sleepadvances/zpag037 (PMC13122618; doi:10.1093/sleepadvances/zpag037)
Supplement: zpag037_Supplemental_Files [file zpag037_supplemental_files.zip › Online_Supplement_zpag037.docx]

**Supplementary materiel**

**A polygenic risk score modifies the cardiovascular risk associated with obstructive sleep apnea**

Christian W. Thorball^1,* ,§^, Adrien Waeber^2,* ,§^, Geoffroy Solelhac^2^, Flavia Hodel^1^, Théo Imler^2^, Grégory Heiniger^2^, Roxane de La Harpe^3^, Pedro Marques-Vidal^3^, Peter Vollenweider^3^, Jacques Fellay^1,4,#^, Raphael Heinzer^2,#^

1 Precision Medicine Unit, Biomedical Data Science Center, Lausanne University Hospital (CHUV) and University of Lausanne, Lausanne, Switzerland.

2 Center for Investigation and Research in Sleep (CIRS), Lausanne University Hospital (CHUV) and University of Lausanne, Lausanne, Switzerland.

3 Department of Medicine, Internal Medicine, Lausanne University Hospital (CHUV) and University of Lausanne, Lausanne, Switzerland.

4 School of Life Sciences, École Polytechnique Fédérale de Lausanne, Lausanne, Switzerland.

* These authors contributed equally

# These authors contributed equally

§ Correspondence: [Christian.Thorball@chuv.ch](mailto:Christian.Thorball@chuv.ch), [Adrien.waeber@chuv.ch](mailto:Adrien.waeber@chuv.ch)

**Table S1. Baseline characteristics according to obstructive sleep apnea status**

|  |  |  | **OSA Status** | | |  | |
| --- | --- | --- | --- | --- | --- | --- | --- |
| **Variable** | **N** | **Overall N = 1,379*** | | **AHI <15 events·h⁻¹ N = 891*** | **AHI ≥15 events·h⁻¹ N = 488*** | | **p-value**** |
| Incident CV event | 1,379 | 100 (7.3%) | | 45 (5.1%) | 55 (11%) | | <0.001 |
| SCORE2 category | 1,379 |  | |  |  | | <0.001 |
| Low risk |  | 768 (56%) | | 592 (66%) | 176 (36%) | |  |
| Moderate risk |  | 472 (34%) | | 245 (27%) | 227 (47%) | |  |
| High risk |  | 139 (10%) | | 54 (6.1%) | 85 (17%) | |  |
| Male | 1,379 |  | |  |  | | <0.001 |
| No |  | 731 (53%) | | 561 (63%) | 170 (35%) | |  |
| Yes |  | 648 (47%) | | 330 (37%) | 318 (65%) | |  |
| Age (y) | 1,379 | 58 (50, 69) | | 55 (48, 67) | 65 (55, 71) | | <0.001 |
| BMI (kg/m2) | 1,373 | 25.6 (23.0, 28.3) | | 24.6 (22.4, 27.1) | 27.3 (25.2, 30.2) | | <0.001 |
| Unknown |  | 6 | | 2 | 4 | |  |
| Current smoker | 1,379 |  | |  |  | | 0.24 |
| No |  | 1,133 (82%) | | 724 (81%) | 409 (84%) | |  |
| Yes |  | 246 (18%) | | 167 (19%) | 79 (16%) | |  |
| Diabetes | 1,379 |  | |  |  | | <0.001 |
| No |  | 1,261 (91%) | | 847 (95%) | 414 (85%) | |  |
| Yes |  | 118 (8.6%) | | 44 (4.9%) | 74 (15%) | |  |
| Systolic BP (mmHg) | 1,379 | 125 (114, 138) | | 122 (111, 135) | 130 (121, 142) | | <0.001 |
| Diastolic BP (mmHg) | 1,379 | 78 (71, 85) | | 77 (70, 84) | 80 (74, 88) | | <0.001 |
| Hypertension | 1,379 |  | |  |  | | <0.001 |
| No |  | 823 (60%) | | 607 (68%) | 216 (44%) | |  |
| Yes |  | 556 (40%) | | 284 (32%) | 272 (56%) | |  |
| Dyslipidemia | 1,379 |  | |  |  | | <0.001 |
| No |  | 1,030 (75%) | | 695 (78%) | 335 (69%) | |  |
| Yes |  | 349 (25%) | | 196 (22%) | 153 (31%) | |  |
| Statins | 1,379 |  | |  |  | | <0.001 |
| No |  | 1,175 (85%) | | 791 (89%) | 384 (79%) | |  |
| Yes |  | 204 (15%) | | 100 (11%) | 104 (21%) | |  |
| HDL Cholesterol (mmol/L) | 1,379 | 1.60 (1.30, 1.90) | | 1.70 (1.40, 2.00) | 1.50 (1.20, 1.80) | | <0.001 |
| Total Cholesterol (mmol/L) | 1,379 | 5.70 (5.00, 6.40) | | 5.70 (5.00, 6.40) | 5.80 (5.10, 6.40) | | 0.071 |
| Weekly alcohol consumption (units) | 1,379 | 4 (1, 10) | | 4 (1, 8) | 6 (2, 12) | | <0.001 |
| AHI (events/h) | 1,379 | 10 (4, 21) | | 6 (3, 9) | 27 (20, 40) | | <0.001 |
| ODI-3% | 1,379 | 10 (4, 19) | | 6 (3, 10) | 25 (18, 37) | | <0.001 |
| Total sleep time (min) | 1,379 | 405 (359, 449) | | 408 (365, 454) | 399 (350, 441) | | 0.003 |
| Sleep efficiency (%) | 1,379 | 88 (80, 93) | | 90 (82, 93) | 84 (75, 91) | | <0.001 |
| Mean SpO2 | 1,379 | 94.20 (93.10, 95.30) | | 94.80 (93.80, 95.60) | 93.40 (92.25, 94.30) | | <0.001 |
| PWADi | 1,369 | 52 (38, 66) | | 52 (38, 65) | 52 (39, 66) | | 0.23 |
| Unknown |  | 10 | | 4 | 6 | |  |

**Table S1 caption:** Baseline characteristics of the 1,379 participants according to obstructive sleep apnea (OSA) status (AHI <15 vs ≥15 events/h)

**Abbreviations:** OSA: obstructive sleep apnea; CV: cardiovascular; SCORE2: Systematic COronary Risk Evaluation 2; BMI: body mass index; BP: blood pressure; HDL: high-density lipoprotein; AHI: apnea–hypopnea index; ODI-3%: oxygen desaturation index (3% desaturation threshold); SpO₂: peripheral oxygen saturation by pulse oximetry; PWADi: pulse wave amplitude drops index.

***** Median (IQR) or frequency (%).

****** P-values derived from Pearson’s chi-squared test for categorical variables or Wilcoxon rank-sum test for continuous variables.

**Table S2. Baseline characteristics according to genetic risk categories and by obstructive sleep apnea status**

|  | **Low Genetic Risk** | | | **Intermediate Genetic Risk** | | | **High Genetic Risk** | | |
| --- | --- | --- | --- | --- | --- | --- | --- | --- | --- |
| **Variable** | **No OSA**  **N = 156*** | **OSA**  **N = 120*** | **p-value**** | **No OSA**  **N = 538*** | **OSA**  **N = 290*** | **p-value**** | **No OSA**  **N = 197*** | **OSA**  **N = 78*** | **p-value**** |
| Incident CV event | 5 (3.2%) | 8 (6.7%) | 0.18 | 20 (3.7%) | 36 (12%) | <0.001 | 20 (10%) | 11 (14%) | 0.35 |
| SCORE2 cat |  |  | <0.001 |  |  | <0.001 |  |  | <0.001 |
| Low risk | 104 (67%) | 49 (41%) |  | 352 (65%) | 99 (34%) |  | 136 (69%) | 28 (36%) |  |
| Moderate risk | 45 (29%) | 48 (40%) |  | 148 (28%) | 140 (48%) |  | 52 (26%) | 39 (50%) |  |
| High risk | 7 (4.5%) | 23 (19%) |  | 38 (7.1%) | 51 (18%) |  | 9 (4.6%) | 11 (14%) |  |
| Sex (Female) | 107 (69%) | 35 (29%) | <0.001 | 319 (59%) | 105 (36%) | <0.001 | 135 (69%) | 30 (38%) | <0.001 |
| Age (y) | 56 (48, 67) | 64 (55, 70) | 0.001 | 55 (48, 67) | 65 (56, 71) | <0.001 | 56 (48, 67) | 62 (55, 70) | <0.001 |
| BMI (kg/m2) | 24.6 (22.2, 26.9) | 26.8 (24.9, 28.4) | <0.001 | 24.3 (22.4, 27.0) | 27.6 (25.5, 30.8) | <0.001 | 25.0 (22.2, 27.6) | 27.6 (24.8, 30.4) | <0.001 |
| *Missing* | *1* | *1* |  | *1* | *2* |  | *0* | *1* |  |
| Current smoker | 25 (16%) | 23 (19%) | 0.49 | 108 (20%) | 43 (15%) | 0.062 | 34 (17%) | 13 (17%) | 0.91 |
| Diabetes | 11 (7.1%) | 12 (10%) | 0.38 | 23 (4.3%) | 53 (18%) | <0.001 | 10 (5.1%) | 9 (12%) | 0.057 |
| Systolic BP (mmHg) | 121 (112, 133) | 130 (119, 141) | <0.001 | 122 (111, 134) | 130 (121, 142) | <0.001 | 123 (111, 136) | 131 (120, 142) | <0.001 |
| Diastolic BP (mmHg) | 76 (69, 83) | 80 (73, 87) | 0.002 | 77 (70, 83) | 81 (74, 88) | <0.001 | 77 (71, 84) | 79 (73, 88) | 0.11 |
| Hypertension | 45 (29%) | 64 (53%) | <0.001 | 168 (31%) | 161 (56%) | <0.001 | 71 (36%) | 47 (60%) | <0.001 |
| Dyslipidemia | 33 (21%) | 40 (33%) | 0.023 | 115 (21%) | 93 (32%) | <0.001 | 48 (24%) | 20 (26%) | 0.83 |
| Statins | 16 (10%) | 18 (15%) | 0.23 | 59 (11%) | 68 (23%) | <0.001 | 25 (13%) | 18 (23%) | 0.033 |
| HDL Cholesterol (mmol/L) | 1.70 (1.40, 2.00) | 1.50 (1.20, 1.70) | <0.001 | 1.70 (1.40, 2.00) | 1.50 (1.20, 1.80) | <0.001 | 1.70 (1.40, 2.00) | 1.50 (1.30, 1.80) | <0.001 |
| Total Cholesterol (mmol/L) | 5.70 (5.10, 6.30) | 5.70 (5.00, 6.30) | 0.47 | 5.60 (5.00, 6.30) | 5.80 (5.10, 6.40) | 0.11 | 5.80 (5.00, 6.50) | 5.90 (5.00, 6.70) | 0.3 |
| Weekly alcohol consumption (units) | 4 (2, 8) | 7 (3, 12) | <0.001 | 4 (1, 9) | 5 (2, 11) | 0.046 | 3 (1, 7) | 5 (1, 10) | 0.058 |
| AHI (events/h) | 6 (3, 10) | 26 (20, 38) | <0.001 | 6 (3, 9) | 27 (19, 41) | <0.001 | 6 (3, 9) | 28 (21, 40) | <0.001 |
| ODI-3% | 5 (3, 10) | 23 (18, 36) | <0.001 | 6 (3, 10) | 25 (18, 37) | <0.001 | 5 (3, 9) | 25 (18, 36) | <0.001 |
| Total sleep time (min) | 407 (370, 450) | 396 (344, 439) | 0.079 | 406 (360, 453) | 397 (350, 440) | 0.032 | 413 (367, 458) | 411 (358, 452) | 0.47 |
| Sleep efficiency (%) | 89 (82, 93) | 84 (75, 91) | 0.001 | 90 (82, 93) | 84 (75, 91) | <0.001 | 90 (82, 93) | 85 (77, 90) | 0.001 |
| Mean SpO2 | 94.75 (93.30, 95.70) | 93.55 (92.50, 94.40) | <0.001 | 94.70 (93.80, 95.60) | 93.30 (92.20, 94.30) | <0.001 | 94.90 (93.90, 95.60) | 93.50 (92.10, 94.40) | <0.001 |
| PWADi | 55 (36, 66) | 52 (39, 67) | 0.77 | 52 (37, 65) | 52 (39, 68) | 0.17 | 52 (39, 65) | 53 (38, 64) | 0.92 |
| *Missing* | *1* | *1* |  | *2* | *3* |  | *1* | *2* |  |

**Table S2 caption:** Baseline characteristics of the 1,379 participants according to genetic risk categories and by obstructive sleep apnea (OSA) status (AHI <15 vs ≥15 events/h)

**Abbreviations:** OSA: obstructive sleep apnea; CV: cardiovascular; SCORE2: Systematic COronary Risk Evaluation 2; BMI: body mass index; BP: blood pressure; HDL: high-density lipoprotein; AHI: apnea–hypopnea index; ODI-3%: oxygen desaturation index (3% desaturation threshold); SpO₂: peripheral oxygen saturation by pulse oximetry; PWADi: pulse wave amplitude drops index.

***** Median (IQR) or frequency (n,%).

****** P-values derived from Pearson’s chi-squared test for categorical variables or Wilcoxon rank-sum test for continuous variables.


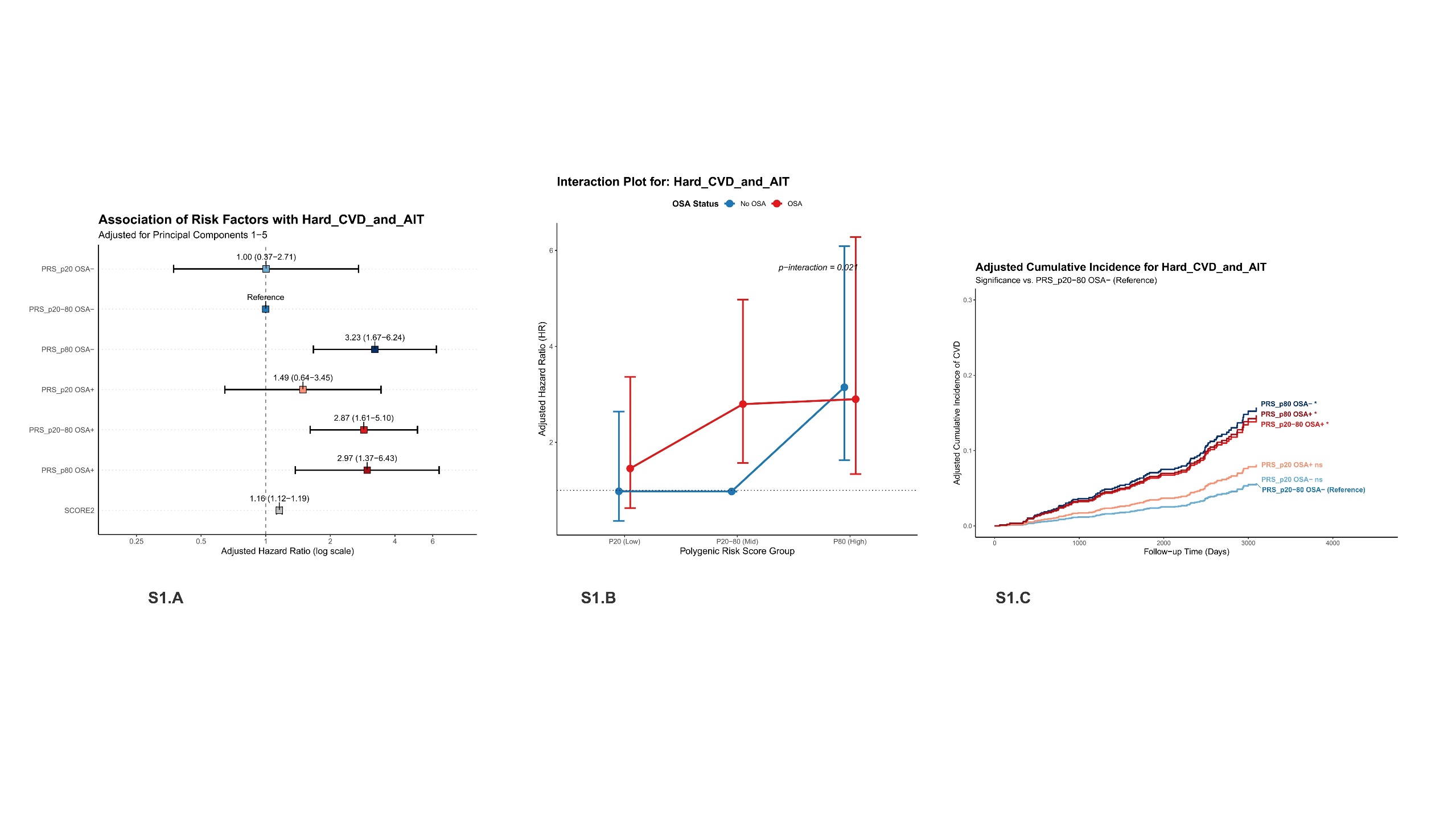
**Figure S1. Interaction between OSA and CAD-PRS for hard cardiovascular outcomes including TIA (A–C)**

**Legend – Figure S1. (**A) Forest plot, (B) interaction plot and (C) cumulative incidence curves for hard CV outcomes including TIA, according to OSA status and CAD-PRS. Hard CV outcomes including TIA comprise CV death, stroke (definite or probable) and TIA (excluding amaurosis fugax and transient global amnesia), AMI and CHD, definite or probable. Models are adjusted for SCORE2/SCORE2-OP; the OSA×PRS interaction is significant (p = 0.021).

**Abbreviations:** OSA, obstructive sleep apnea; CAD-PRS, coronary artery disease polygenic risk score; PRS, polygenic risk score; CV, cardiovascular; TIA, transient ischaemic attack; AMI, acute myocardial infarction; CHD, coronary heart disease

**
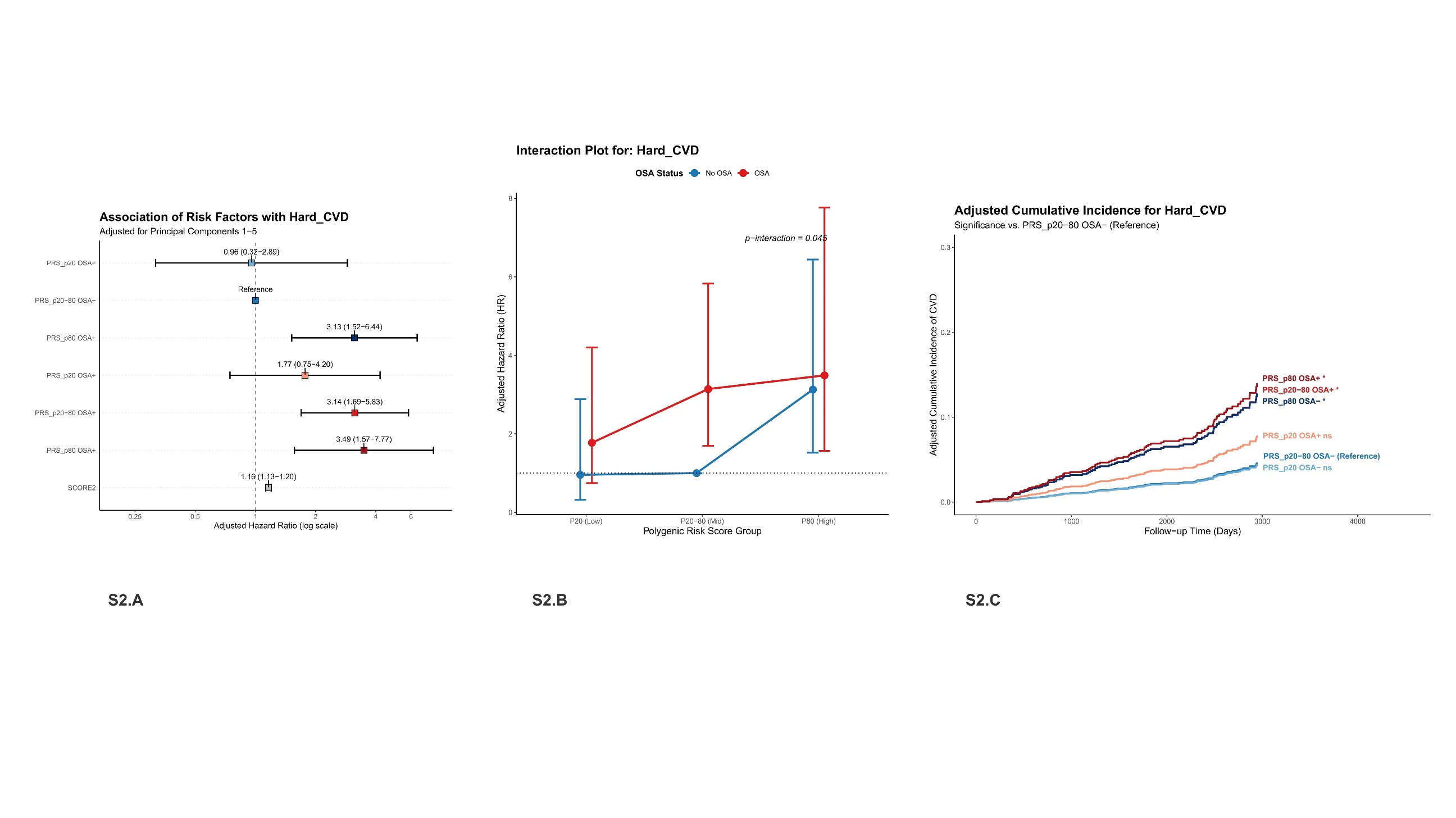
Figure S2. Interaction between OSA and CAD-PRS for hard cardiovascular outcomes excluding TIA (A–C)**

**Legend – Figure S2.** (A) Forest plot, (B) interaction plot and (C) cumulative incidence curves for hard CV outcomes excluding TIA, according to OSA status and CAD-PRS. Hard CV outcomes excluding TIA comprise cardiovascular death, stroke (definite or probable; excluding TIA, amaurosis fugax and transient global amnesia), AMI and CHD, definite or probable. Models are adjusted for SCORE2/SCORE2-OP; the OSA×PRS interaction remains significant (p-value = 0.045).

**Abbreviations:** OSA, obstructive sleep apnea; CAD-PRS, coronary artery disease polygenic risk score; PRS, polygenic risk score; CV, cardiovascular; TIA, transient ischaemic attack; AMI, acute myocardial infarction; CHD, coronary heart disease

**
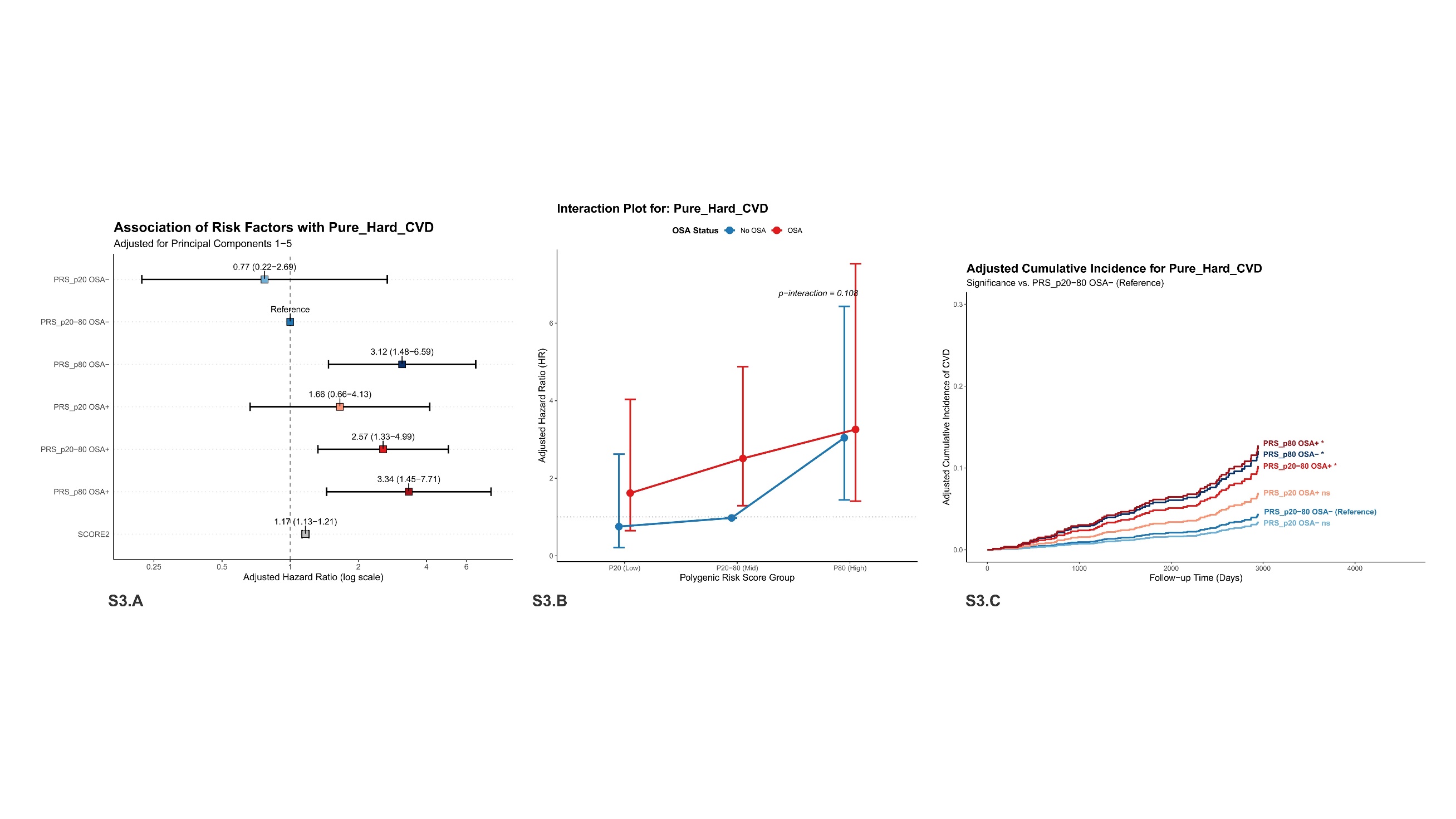
Figure S3. Interaction between OSA and CAD-PRS for pure hard cardiovascular outcomes (A–C)**

**Legend – Figure S3.** (A) Forest plot, (B) interaction plot and (C) cumulative incidence curves for pure hard CV outcomes according to OSA status and CAD-PRS. Pure hard CV outcomes comprise cardiovascular death, definite stroke, definite AMI and definite CHD. Models are adjusted for SCORE2/SCORE2-OP; the OSA×PRS interaction shows a similar trend to the main analysis (p-value = 0.108).

**Abbreviations:** OSA, obstructive sleep apnea; CAD-PRS, coronary artery disease polygenic risk score; PRS, polygenic risk score; CV, cardiovascular; AMI, acute myocardial infarction; CHD, coronary heart disease

**
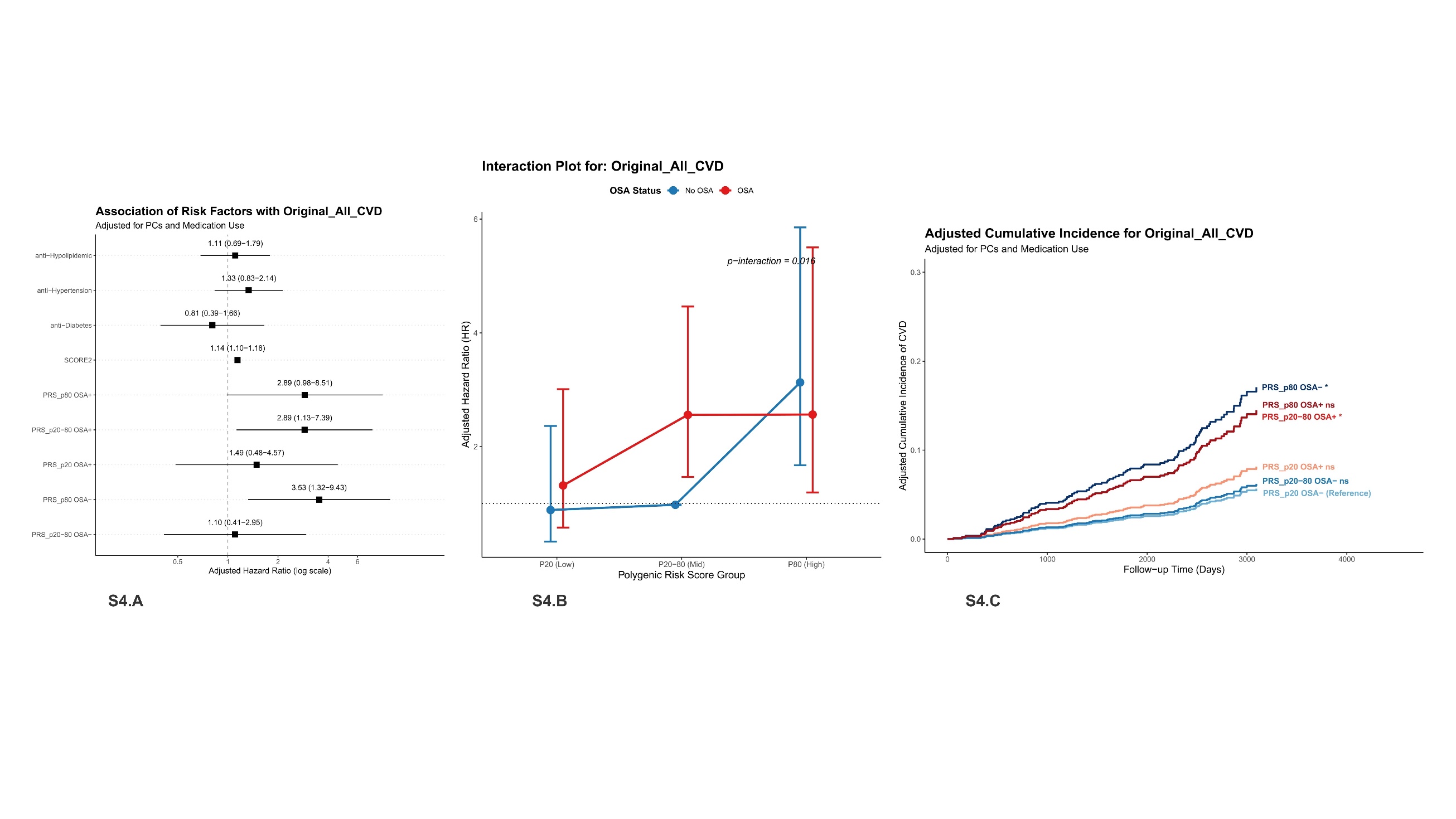
Figure S4. Interaction between OSA and CAD-PRS for any cardiovascular disease after adjustment for medication (A–C)**

**Legend – Figure S4.** (A) Forest plot, (B) interaction plot and (C) cumulative incidence curves for any CVD according to OSA status and CAD-PRS, with additional adjustment for antidiabetic, antihypertensive and lipid-lowering therapies. Models are adjusted for SCORE2/SCORE2-OP and medication use; the OSA×PRS interaction remains significant (p-value = 0.016).

**Abbreviations:** OSA, obstructive sleep apnea; CAD-PRS, coronary artery disease polygenic risk score; PRS, polygenic risk score; CV, cardiovascular

**
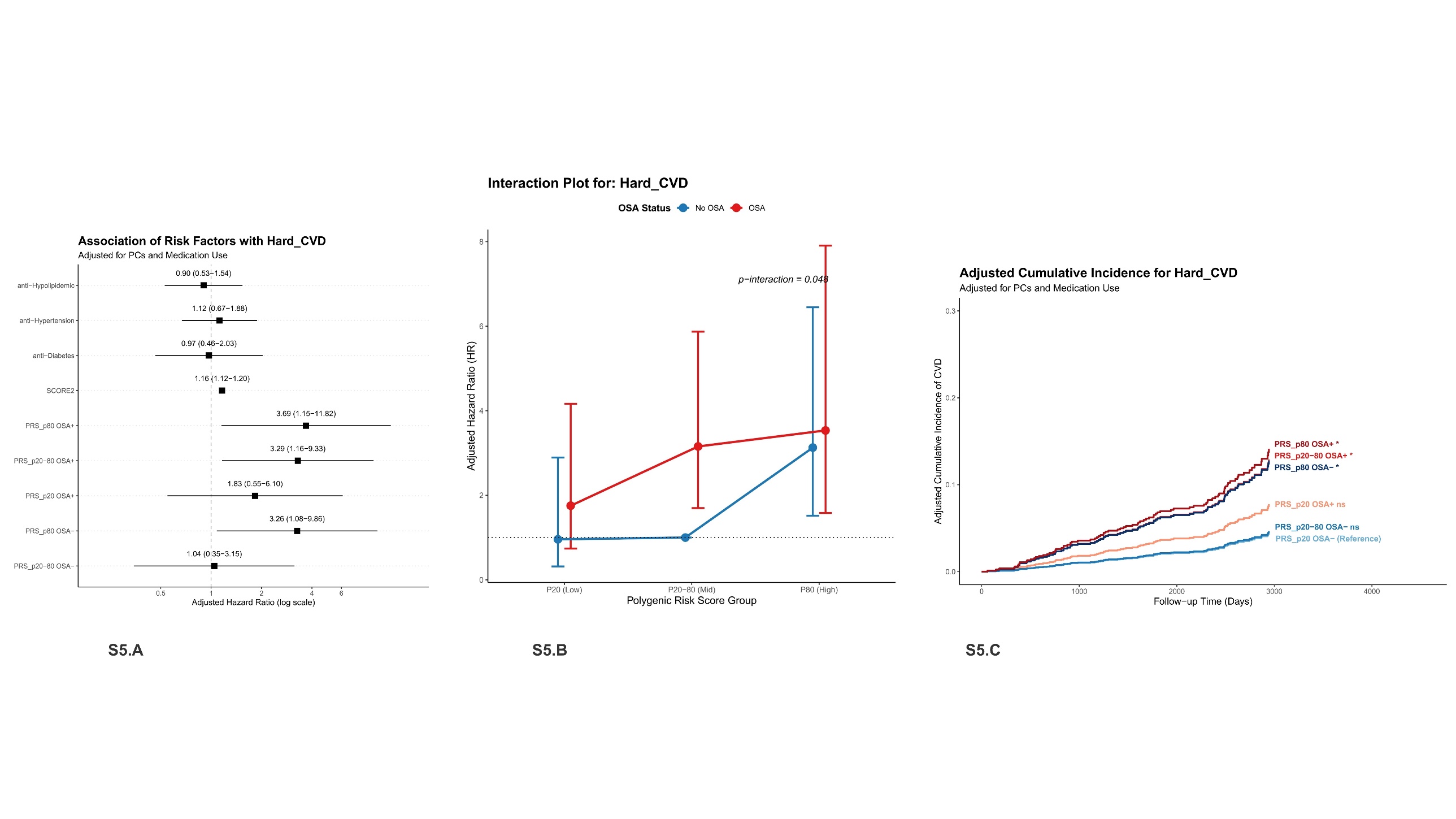
Figure S5. Interaction between OSA and CAD-PRS for hard cardiovascular disease after adjustment for medication (A–C)**

**Legend – Figure S5.** (A) Forest plot, (B) interaction plot and (C) cumulative incidence curves for hard CVD according to OSA status and CAD-PRS, further adjusted for antidiabetic, antihypertensive and lipid-lowering therapies. Hard CVD is defined as in Figure S2. Models are adjusted for SCORE2/SCORE2-OP and medication use; the OSA×PRS interaction remains significant (p-value = 0.048).

**Abbreviations:** OSA, obstructive sleep apnea; CAD-PRS, coronary artery disease polygenic risk score; PRS, polygenic risk score; CVD, cardiovascular disease; AMI, acute myocardial infarction; CHD, coronary heart disease

**
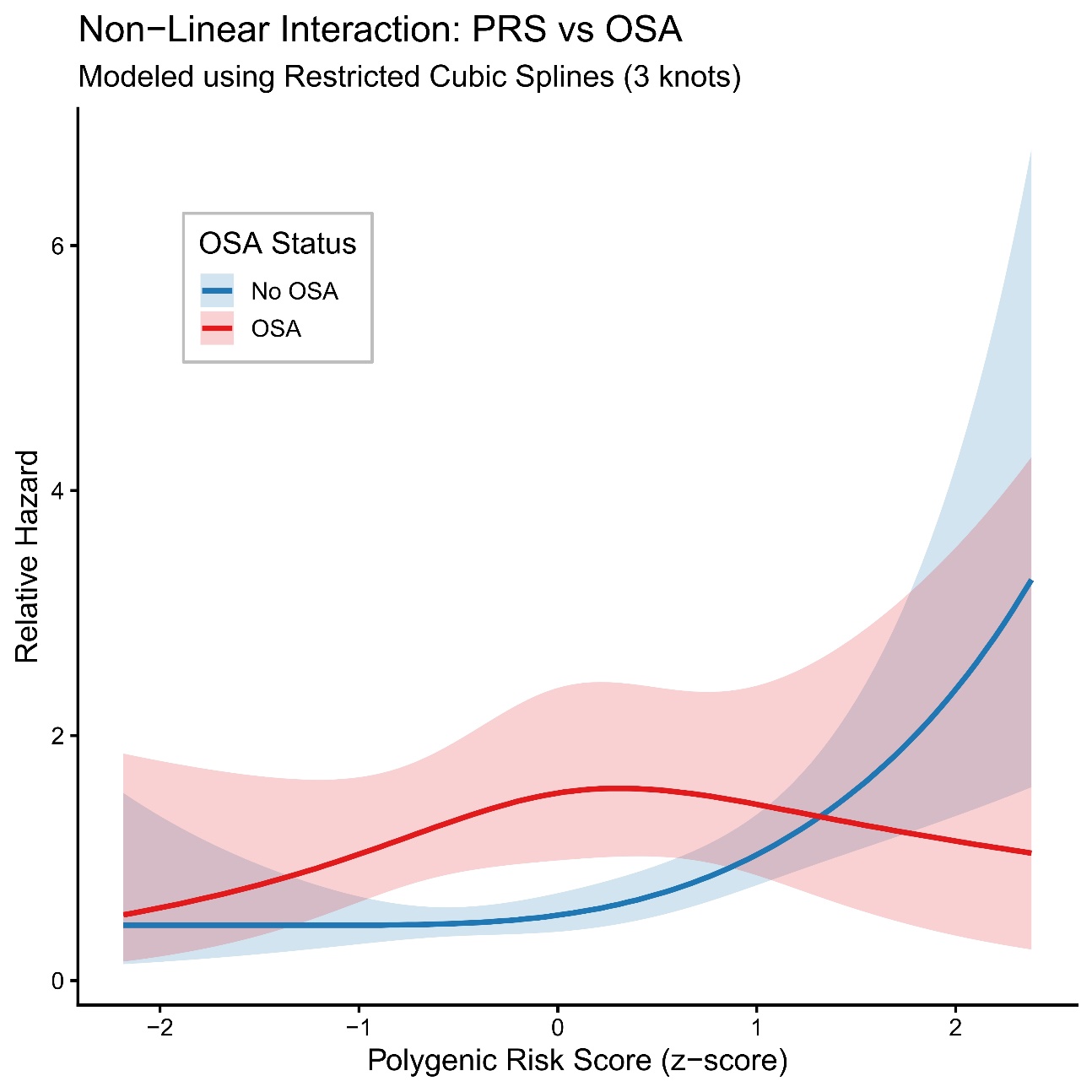
Figure S6. Non-linear interaction between polygenic risk and OSA status.**

**Legend – Figure S6:** Restricted cubic spline model (3 knots) was used to estimate the relative hazard across the polygenic risk score (PRS; z-score), stratified by OSA status. Solid lines represent point estimates and shaded areas indicate 95% confidence intervals. The curves show a non-linear pattern with distinct risk divergence in the intermediate PRS range, supporting effect modification of genetic susceptibility by OSA status.

**
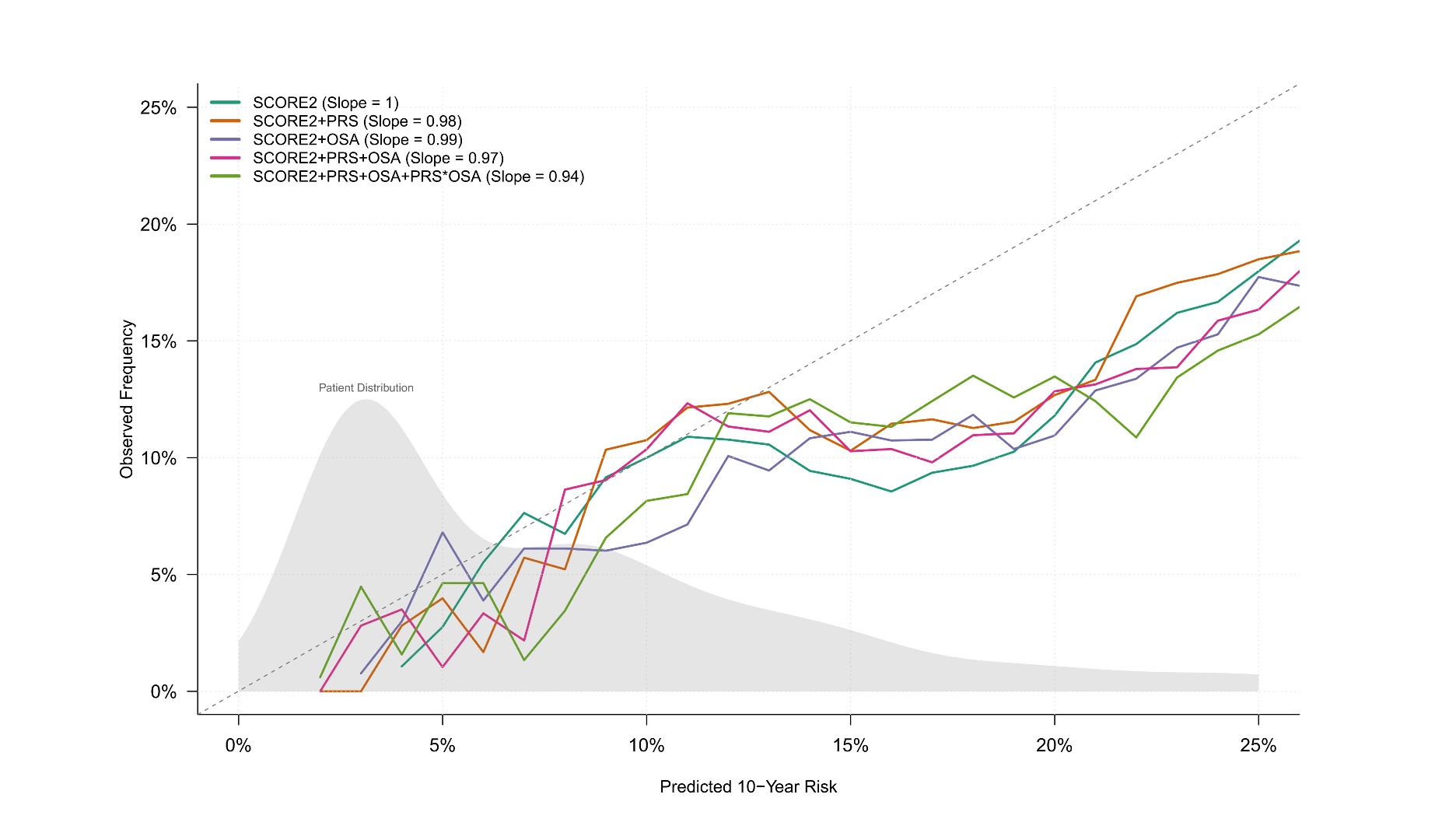
Figure S7. Calibration of models for 10-year cardiovascular risk**

**Legend – Figure S7.**The plot illustrates the agreement between predicted 10-year risk (x-axis) and observed event frequency (y-axis). The diagonal dashed line represents perfect calibration. The gray shaded area depicts the distribution of predicted risks within the cohort, showing that the vast majority of participants fall into the low-to-intermediate risk category (<10%). All models showed good calibration, with predicted risks closely matching the ideal line of perfect agreement in the low and intermediate risk ranges, indicating no systematic bias in risk estimation. In the high-risk range (>10%), models tended to overestimate risk, reflecting the sparsity of data in the upper tail and the potential impact of preventive interventions (e.g., lipid-lowering therapy) initiated in high-risk individuals during the follow-up period.

Abbreviations: OSA, obstructive sleep apnea; PRS, polygenic risk score.
