# Supplementary material for: A polygenic risk score modifies the cardiovascular risk associated with obstructive sleep apnea
Source: Sleep Adv. 2026 Mar 23;7(2):zpag037. doi: 10.1093/sleepadvances/zpag037 (PMC13122618; doi:10.1093/sleepadvances/zpag037)
Supplement: zpag037_Supplemental_Files [file zpag037_supplemental_files.zip › OSAxPRS_graphical_abstract_V1_zpag037.pdf]

How does a Polygenic Risk Score help to assess and reclassify cardiovascular risk in Obstructive Sleep Apnea patients beyond standard risk scores?

Abbreviations
AHI – Apnea/hypopnea index
CAD – Coronary Artery Disease
CV – Cardiovascular
OSA – Obstructive Sleep Apnea
PRS – Polygenic Risk Score

Methods

Baseline
Complete clinical assessment, genotyping and full polysomnography

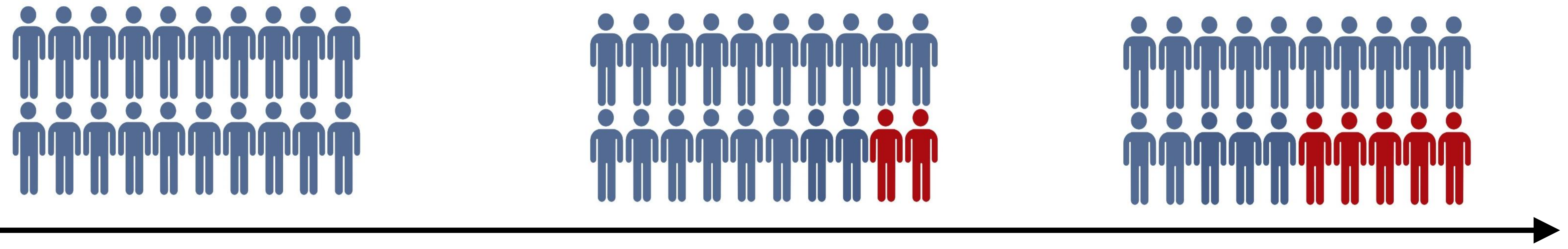

1379 participants
nested within the CoLaus|HypnoLaus Swiss-based population cohort
Follow-up of **10.8 years (median 7.2 years)**
Number of CVD: **100**

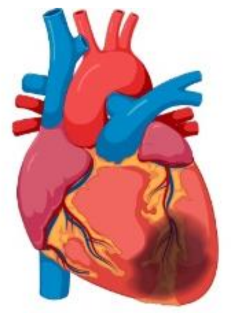

CV Outcomes

Stroke
Acute myocardial infarction
Coronary heart disease
CV death

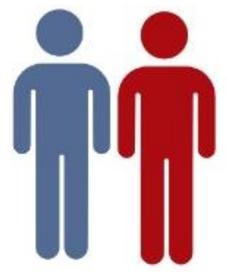

Sample

Median age [IQR]: **58 [50-69] years**
Median BMI [IQR]: **25.6 [23-28.3] kg/m²**
OSA/non-OSA patients: **35/65%**
Women/men: **53/47%**

1 OSA Status

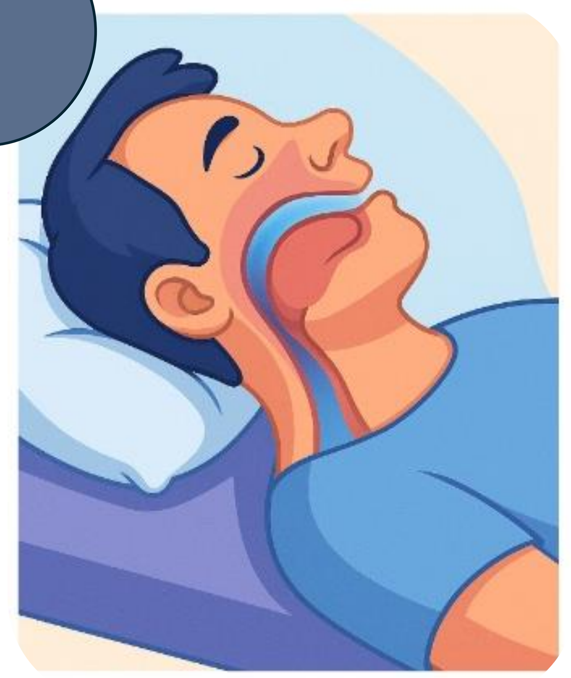

OSA – AHI ≥ 15/h
Non-OSA – AHI <15/h

- Sex
- Age
- Smoking Status
- Systolic blood pressure
- Total- and HDL-Cholesterol
- Diabetes (SCORE2-OP)

2 SCORE2-(OP) Risk Score

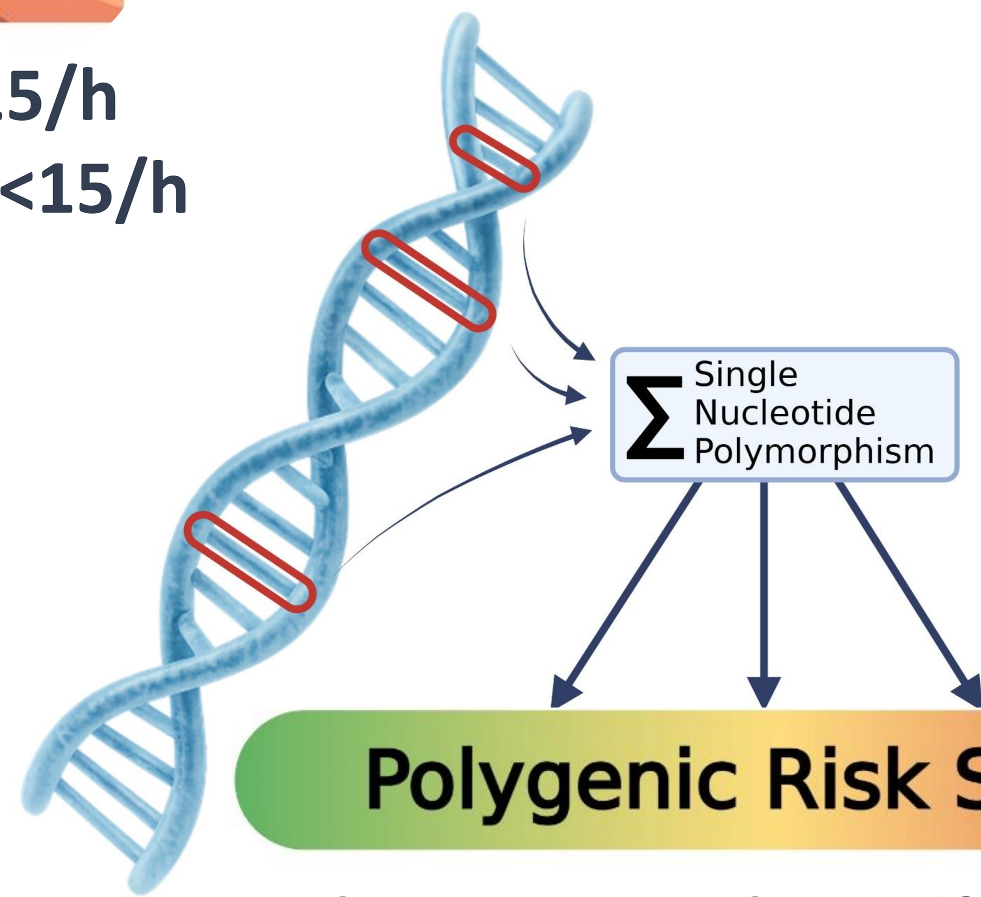

3 Polygenic Risk Score

CAD-PRS by Inouye et al. stratified into three groups: low (P0–20), intermediate (P20–80), high (P80–100)

Association between OSA and CV Events by PRS categories

Cox models adjusted for SCORE2/SCORE2-OP

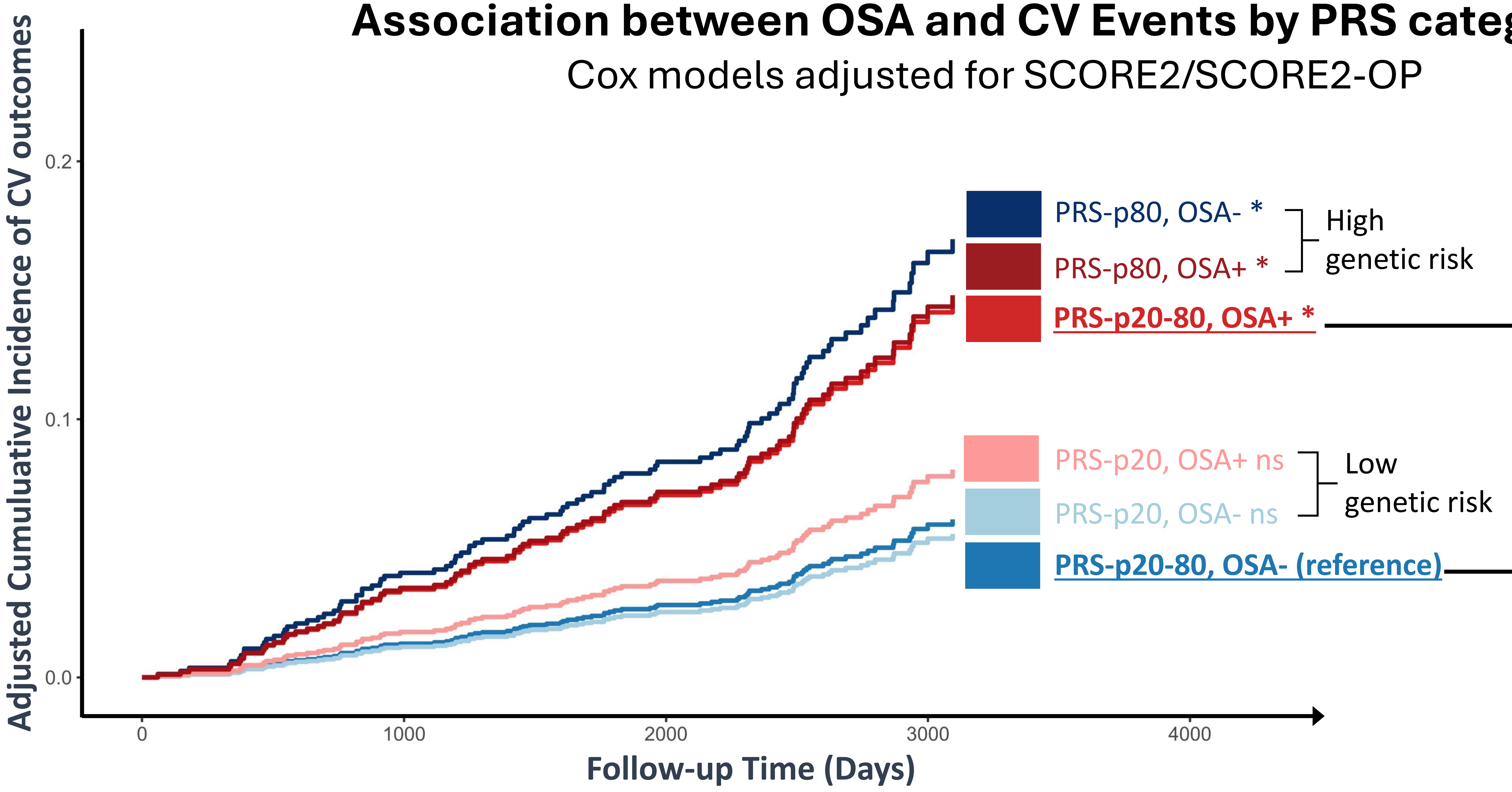

OSA x PRS interaction p-value = 0.013

A CAD-PRS refines CV risk stratification in OSA, especially in individuals with intermediate genetic risk where the CV risk associated with OSA is the highest. Incorporating genetic risk and OSA status into established clinical scores such as SCORE2 significantly improved model performance and reclassification.
